# Combining Oxygen-Enhanced MRI and Electron Paramagnetic Resonance Oximetry for Quantitative OE-MRI (qOE-MRI) as a Method for Mapping Absolute Oxygen Levels (pO_2_) in Tumors

**DOI:** 10.1002/mrm.70418

**Published:** 2026-05-10

**Authors:** Conner S. Ubert, Victor B. Kassey, Maciej M. Kmiec, Diana J. Wallin, Alireza Kheirollah, Sergey V. Petryakov, Ryan C. O’Connell, Philip E. Schaner, Periannan Kuppusamy

**Affiliations:** 1 Department of Radiology, Geisel School of Medicine, Dartmouth College, Hanover, New Hampshire, USA; 2 Thayer School of Engineering, Dartmouth College, Hanover, New Hampshire, USA; 3 Department of Psychiatry, Geisel School of Medicine, Dartmouth College, Hanover, New Hampshire, USA; 4 Department of Surgery, Geisel School of Medicine, Dartmouth College, Hanover, New Hampshire, USA; 5 Department of Radiation Oncology and Applied Sciences, Geisel School of Medicine, Dartmouth College, Hanover, New Hampshire, USA

**Keywords:** EPR oximetry, OE-MRI, OxyChip, SCC7 tumor, tumor hypoxia

## Abstract

**Purpose:**

Tumor hypoxia remains a major barrier to effective radiation therapy, yet no current imaging technique can generate spatially resolved, absolute pO_2_ maps with sufficient depth penetration and spatial resolution for clinical use. This study introduces quantitative oxygen-enhanced MRI (qOE-MRI), a multimodal approach integrating electron paramagnetic resonance (EPR) oximetry using the OxyChip with oxygen-enhanced MRI to enable high-resolution mapping of absolute tumor pO_2_.

**Methods:**

Phantom studies at 9.4 T validated the linearity of the R_1_–pO_2_ relationship using saturation-recovery T_1_ mapping across known oxygen concentrations. OxyChip sensors coated with gold nanoparticles were used for EPR oximetry. *In vivo* qOE-MRI was evaluated and tested in an SCC7 mouse tumor model by combining L-band EPR oximetry under normoxic and hyperoxic breathing with quantitative T_1_ mapping at 9.4 T. Tissue-specific R_1_ versus pO_2_ calibration was established using EPR-derived pO_2_ measurements at implanted OxyChip locations and applied voxel-wise to generate spatial pO_2_ maps.

**Results:**

Phantom validation demonstrated excellent R_1_–pO_2_ linearity (*R*^2^ = 0.999) with predictive precision of 2.6 mmHg. OxyChip characterization confirmed robust oxygen sensitivity (12.6 mG/mmHg) and MRI visibility with gold coating. *In vivo* calibration showed a strong R_1_ –pO_2_ correlation (*R*^2^ = 0.9737). Resulting pO_2_ maps revealed marked intratumoral heterogeneity: the hypoxic core on average showed no increase in oxygenation due to hyperoxygenation, whereas peripheral and muscle-adjacent regions exhibited increased oxygenation.

**Conclusion:**

qOE-MRI enables repeatable and spatially resolved absolute tumor pO_2_ mapping. By combining OxyChip technology, clinical EPR systems, and standard MRI platforms, this approach holds promise for translation into hypoxia-guided radiation therapy planning.

## 1 | Introduction

Hypoxia is a well-characterized feature of the solid tumor microenvironment and one of the most notable barriers to successful cancer treatment [1–4]. As tumors grow, oxygen demand progressively exceeds supply due to rapid cellular proliferation outpacing vascular development, elevated metabolic activity, vascular dysfunction, and necrosis—collectively producing regions unreachable by oxygen diffusion [5–9]. The culmination of this heterogeneous tumor oxygenation is that hypoxia becomes variable not only spatially across the tumor volume but also temporally as vascular blood flow fluctuates, making it difficult to track [10, 11].

Among other reasons, tumor hypoxia is clinically significant in that it diminishes the efficacy of radiation therapy. Ionizing radiation generates reactive oxygen species (ROS) that damage DNA. Molecular oxygen then stabilizes this damage by reacting with DNA radicals, converting them to peroxyl radicals and preventing repair [5, 12]. In hypoxic cells, oxygen fixation does not occur, and DNA is more readily repaired. The oxygen enhancement ratio (OER) expresses this relationship between oxygen and radiation-induced cell kill, stating that cells with very low oxygen (< 10 mmHg) need about two to three times the radiation dose to reach the same biological effect as cells with normal oxygen levels [5, 13, 14]. Consequently, radiobiologically significant hypoxia is strongly associated with increased local recurrence and distant metastases [15–18]. These data have driven interest in hypoxia-directed treatment strategies, including dose escalation to hypoxic sub-volumes and the use of radiosensitizing agents, both of which require reliable spatial knowledge of hypoxic regions within the tumor [18–21].

Reliable mapping of the intratumoral partial pressure of oxygen (pO_2_) is therefore a critical unmet need in radiation oncology. Electron paramagnetic resonance (EPR) oximetry is a well-established technique for measuring tissue pO_2_ *in vivo* [22–25]. EPR spectroscopy uses the interaction between an applied magnetic field and unpaired electrons in paramagnetic species to generate a characteristic absorption spectrum [23]. OxyChip is an implantable EPR oxygen sensor that utilizes this mechanism by combining the oxygen-permeable polymer polydimethylsiloxane (PDMS) with the EPR spin probe lithium octa-n-butoxy-naphthalocyanine (LiNc-BuO) [26–28]. The LiNc-BuO crystal is a molecule with unpaired electrons (paramagnetic) that produces an EPR signal proportional to the presence of oxygen. The linewidth (magnetic field separation from peak-to-peak) of the EPR signal has a linear relationship with adjacent tissue pO_2_, enabling the precise quantification of local oxygen levels [29, 30].

OxyChip is a flexible semi-solid that has been shown to be stable in tissue for at least 1 year after implantation [31]. OxyChip has been successfully used for *in vivo* oximetry in several pre-clinical models and has been used in a first-in-human clinical trial in cancer patients [27, 32–34]. Key attributes of OxyChip include high oxygen sensitivity, biocompatibility, FDA Investigational Device Exemption (IDE) for long-term implantation, multimodal visibility, signal stability over time, and a linear response across physiologically relevant oxygen ranges.

Despite the high accuracy of EPR spectroscopic oximetry, the technique of using implantable sensors is inherently limited to point measurements at discrete locations, providing no information about the spatial distribution of pO_2_ across the larger tissue volume. Oxygen-enhanced MRI (OE-MRI) resolves this limitation by exploiting the paramagnetic relaxation effect of dissolved molecular oxygen on tissue water protons [35–38]. Molecular oxygen, being paramagnetic, acts as an endogenous contrast agent that shortens the longitudinal relaxation time (T_1_) of surrounding water protons. This effect presents as an increase in the longitudinal relaxation rate R_1_ (1/T_1_) that is linearly proportional to the local pO_2_ [39–41].

OE-MRI protocols typically exploit this relationship by acquiring T_1_ or R_1_ maps under two breathing conditions with a baseline normoxic state (e.g., 21% O_2_) and a hyperoxic challenge (e.g., 100% O_2_) and comparing the resulting maps to identify regions of tissue that actively take up (or respond to) the additional dissolved oxygen [42–44]. Tissues that are well-vascularized and capable of absorbing oxygen show a measurable increase in R_1_ under hyperoxic conditions, while poorly vascularized or hypoxic regions show little to no response, or in some cases, a negative response [43]. However, OE-MRI only measures relative changes in R_1_ in different breathing conditions and does not directly report absolute pO_2_ values. This is because the R_1_ signal in tissue is affected by multiple factors in addition to oxygen, including macromolecular content, cellular density, and pH, which means that converting measured R_1_ to pO_2_ requires a tissue-specific calibration reference. In this study, we combine OE-MRI with EPR oximetry to overcome the principal limitation of each technique: OE-MRI provides spatially resolved maps of tissue oxygen response but no direct measure of absolute pO_2_, while EPR spectroscopy with implanted OxyChips yields accurate, absolute pO_2_ measurements at discrete locations but no volumetric information. Together, these modalities enable quantitative, three-dimensional mapping of tumor pO_2_.

## 2 | Methods

### 2.1 | Phantom Study

#### 2.1.1 | Phantom Preparation

To verify the linear relationship between R_1_ and pO_2_ at 9.4 T, six phantoms filled with deionized water were prepared with known O_2_ concentrations. The phantoms were individually equilibrated with compressed gas mixtures (Airgas, Radnor, PA) containing 0%, 2%, 5%, 15%, 21%, and 30% O_2_ for 30 min. The oxygenated water was immediately transferred to 5-mm outer diameter standard series NMR tubes, capped, and sealed with polyolefin paraffin wax film. Samples were positioned in a custom cylindrical holder (diameter = 29.75 mm) radially equidistant from the coil center. Imaging took place within 20 min of sealing. Given the short elapsed time between sealing and acquisition, any diffusive loss or gain of dissolved O_2_ was considered inconsequential.

#### 2.1.2 | MRI Protocol for Phantom Validation

All phantom measurements were performed using a 9.4 T pre-clinical MRI system (Varian magnet with Bruker upgrade, Bruker BioSpin, Billerica, MA). A custom-made ceramic volume coil (inner diameter = 30 mm, length = 43 mm) tuned to 400.5 MHz was used as the receive coil, while the stock Bruker body coil was used for transmission [45]. T_1_ mapping was performed using a variable repetition time (TR) saturation recovery sequence based on a Rapid Acquisition with Relaxation Enhancement (RARE) sequence. The saturation recovery method was chosen over inversion recovery to reduce scan time and sensitivity to motion-induced artifacts when transitioning to *in vivo* applications. The sequence parameters were as follows: sequence type = RARE 2D acquisition, echo time (TE) = 19.6 ms, TR = (355, 528, 716, 925, 1158, 1422, 1725, 2084, 2520, 3080, 3860, 5155, and 10 000 ms), echo train length (ETL) = 5, slice thickness = 2 mm, slice gap = 0.3 mm, flip angle (FA) = 90°, averages (AVG) = 2, FOV= 30 × 30 mm^2^, matrix size = 256 × 256, in-plane resolution = 0.117 × 0.117 mm^2^, receiver bandwidth (BW) = 318 Hz/pixel. Each T_1_ mapping acquisition required approximately 15 min. The average signal intensity for each phantom tube is fitted to the longitudinal relaxation function to determine T_1_ and R_1_.

### 2.2 | OxyChip Preparation and Characterization

#### 2.2.1 | OxyChip Fabrication, Characterization, and Sterilization

OxyChip is an implantable EPR oxygen sensor capable of accurately measuring tissue pO_2_. OxyChip combines PDMS and the EPR spin probe, LiNc-BuO, to form a biocompatible material for measuring tissue oxygenation. The OxyChips were fabricated in-house. To enhance imaging visibility, gold nanoparticles (GNPs) were incorporated onto the OxyChip surface using the method described by Kmiec et al. [46] LiNc-BuO crystal morphology was characterized using scanning electron microscopy. The visibility of GNP-incorporated OxyChips for localization was evaluated using a Quantum GX3 micro-CT scanner (Revvity, Hopkinton, MA). The OxyChips were sterilized before calibration and *in vivo* use. See Supporting Information for additional details.

#### 2.2.2 | EPR Calibration of OxyChips

The OxyChips were calibrated to establish the relationship between EPR spectral linewidth and pO_2_. One OxyChip from the batch was placed in a sealed calibration chamber with gas inlet and outlet ports. The chamber was filled with gas containing 0%–30% O_2_ for 5 min at each concentration. Gas concentrations were verified using the FD02 optical oxygen sensor positioned in-line with the chamber inlet. EPR spectra were acquired at each oxygen concentration over 12 averages (see Supporting Information for spectrometer details). The peak-to-peak linewidth was determined using custom software on the spectrometer. Linear regression yielded the calibration equation: pO_2_ (mmHg) = m • linewidth + b, where “m” is the sensitivity of the OxyChip, and “b” is the anoxic linewidth.

### 2.3 | *In Vivo* qOE-MRI Studies

This study presents a proof-of-concept of the qOE-MRI workflow; full results are reported for one animal with the highest calibration point count, with supporting data from a second animal provided in the Supporting Information.

#### 2.3.1 | Animal Model

All animal procedures used in this study were approved by the Institutional Animal Care and Use Committee (IACUC) at Dartmouth and conducted in accordance with the Guide for the Care and Use of Laboratory Animals. Male C3H mice (6–8 weeks old, 20–35 g) were used to validate this procedure (Jackson Laboratories, Bar Harbor, ME). Mice were anesthetized with 1.5% isoflurane (induced at 3%) in 21% O_2_ through a nose cone, with the thigh area shaved (2×2 cm^2^) and sterilized with betadine solution and 70% alcohol swab. The mice were intramuscularly injected (in the right hind leg) with 1×10^6^ SCC7 (squamous cell carcinoma) cells in 100 μL of tumor development medium. Tumor volumes were monitored using a digital caliper. The tumor volume was determined using the formula: V = π/6 • width • length • height.

#### 2.3.2 | OxyChip Implantation

Mice were anesthetized with isoflurane (3% induction, 1.5%–2.0% maintenance) with body temperature maintained at 37°C with a heat pad and respiratory rate at 60–90 breaths/min by modulating isoflurane. Three sterilized OxyChips were implanted throughout the tumor using the pre-loaded needles and stylets. Additionally, a control injection was made that did not contain an OxyChip to assess tissue response post-injection. A 3-day recovery period was observed to allow for tissue stabilization and to resolve any implantation-related inflammation before imaging.

#### 2.3.3 | Micro-CT Localization of OxyChips

Before EPR measurements, mice were imaged using the Quantum GX3 micro-CT scanner with an isotropic image resolution of 25.75 μm to determine the three-dimensional positions of the implanted OxyChips within the tumor. CT images were imported into a 3D imaging software (Dragonfly 3D, Montreal, CA) for analysis. OxyChips appeared as high-attenuation objects (bright regions) due to the gold nanoparticle coating, providing a clear contrast against the soft tissue. The 3D positions of each OxyChip were used to guide EPR resonator placement.

#### 2.3.4 | EPR Oximetry Measurements

The mice were then placed in a continuous-wave (CW) L-band EPR spectrometer while breathing 21% O_2_ (anesthesia: 1.5% isoflurane, induced with 3% isoflurane) through a nose cone at 1.0 L/min and kept at 37°C with a heat lamp and heat pad. EPR measurements were performed on a custom L-band spectrometer (see Supporting Information for hardware details); sweep time was 6 s per scan with 50 averages. The surface loop resonator was positioned over the tumor and carefully placed to avoid potential overlapping OxyChip signals. The breathing gas was then swapped to 100% O_2_ and maintained for 20 min before the measurements were repeated. Total time was approximately 50 min. Mice were maintained prone throughout to permit resonator contact and ensure consistency across modalities.

#### 2.3.5 | OE-MRI Measurements

Following EPR measurements, the mice were allowed to recover from anesthesia for 2 h at room temperature. Mice were monitored until they exhibited normal grooming behavior. The MRI measurements were performed on the same 9.4T pre-clinical MRI system used for phantom imaging described in Section 2.1.2. The mice were anesthetized with isoflurane (3% induction, 1.5% maintenance) while breathing 21% O_2_ at 1.0 L/min and kept at 37°C using heated forced air. Body temperature was maintained throughout the experiment to minimize R_1_ fluctuations from thermal drift between acquisitions. The mice were positioned prone in the ceramic volume coil. Respiratory rate was monitored using a pressure sensor and maintained at 60–90 breaths/min. A series of saturation-recovery relaxometry sequences were performed to map tissue T_1_ while the mouse was breathing 21% O_2_. The sequence parameters were as follows: sequence type = RARE, 2D acquisition, TE = 19.6 ms, TR= [868, 971, 1087, 1217, 1368, 1545, 1760, 2035, 2415, 3035, and 5000 ms], ETL = 6, slice thickness = 0.7 mm, slice gap = 0.1 mm, FA = 90°, AVG = 1, FOV= 60 × 60 mm^2^, matrix size = 256 × 256, in-plane resolution = 0.234 × 0.234mm^2^, BW= 318 Hz/px. The breathing gas was swapped to 100% O_2_ and maintained for 20 min at which point both T_1_-weighted (TR/TE = 1500/8 ms, ETL =4, AVG = 2, FA = 90°) and intermediate T_2_-weighted (TR/TE = 2610/53 ms, ETL = 8, AVG = 2, FA = 90°C) anatomical images were collected, before repeating the T_1_ mapping protocol. Total scan time was approximately 65 min, including the 20-min gas equilibration period.

### 2.4 | Image Processing and Data Analysis

#### 2.4.1 | Phantom Data Analysis

DICOM images were imported into ImageJ for analysis. Circular regions of interest (ROIs, diameter = 3.6 mm, ~750 px per ROI) were defined at the center of each phantom tube. The ROIs were chosen to be smaller than the tube to avoid edge artifacts. ROI positions were applied consistently across all variable TR images. Voxel-wise T_1_ maps were generated using the Bruker T1sat saturation recovery fitting protocol in ParaVision 6.0.1 and converted to R_1_ maps (R_1_ = 1/T_1_).

These R_1_ maps were used for subsequent analysis and to establish the linear relationship between R_1_ and pO_2_. Mean ROI R_1_ values were plotted against pO_2_ to determine the linear relationship R_1_ = R_1,0_ + r_1_•pO_2_, where R_1,0_ is the anoxic baseline and r_1_ is the relaxivity. The correlation was assessed using Pearson’s correlation coefficient, and the statistical significance of the linear relationship was determined using an *F*-test on the regression model.

#### 2.4.2 | EPR Data Analysis

The raw EPR spectra were exported and processed using a custom MATLAB script. Spectra were first denoised using the wavelet denoising method described by Srivastava et al. [47] Following denoising, the first-derivative peak-to-peak linewidth was determined by identifying the two extrema of the derivative spectrum and calculating the magnetic field difference between them. The linewidth values (milligauss, mG) were converted to pO_2_ (mmHg) using the batch-specific calibration equation determined in Section 2.2.2. The mean pO_2_ across all resolved OxyChip signals was calculated and used as the reference value to relate to the MRI-derived R_1_ measurements.

#### 2.4.3 | *In Vivo* MRI Data Analysis

*In vivo* DICOM images were processed using a workflow similar to the phantom data. The tumor ROIs were manually segmented from T_1–_ and T_2_-weighted anatomical images. The OxyChip locations were identified on the anatomical images as small signal voids with sections of nearby brightness caused by susceptibility artifacts from the GNPs. The sub-ROIs were defined as the voxels within 300 μm of each OxyChip that were not impacted by susceptibility artifacts. Maps of relaxation rate were generated by taking the reciprocal of the voxel-wise fitting result of the saturation recovery analysis (same equation as Section 2.4.1).

#### 2.4.4 | Generation of Quantitative pO_2_ Maps

For each OxyChip, the mean effective spin–lattice relaxation rate values (R_1_) were extracted from the sub-ROIs around each OxyChip under both 21% O_2_ and 100% O_2_ breathing conditions. The R_1_ values, a result of the multi-echo spin–lattice relaxometry sequence, were correlated with the corresponding EPR-measured pO_2_ values obtained under identical gas-breathing conditions at the same locations. The multi-echo acquisition introduces R_2_-dependent modulation of the apparent R_1_ maps, meaning the measured R_1_ reflects both longitudinal relaxation and transverse relaxation contributions unique to the tissue environment. Consequently, the simultaneous acquisition of pO_2_ and apparent R_1_ under these *in vivo* conditions effectively yields a subject-specific calibration curve [48]. The calibration equation was then applied voxel-wise to the entire tissue R_1_ map to generate a quantitative pO_2_ map. Median and top-hat filters were applied to reduce noise and large background variations. The hypoxic fraction (hypoxic percentage) was calculated as the percentage of tumor voxels with pO_2_ < 10 mmHg [49]. Three-dimensional pO_2_ distributions were rendered using either ImageJ projections or a custom MATLAB script.

### 2.5 | Statistical Analyses

All statistical analyses were performed in MATLAB. Correlation for the OxyChip calibration curve and the R_1_–pO_2_ relationship was determined using Pearson’s correlation. The two-tailed *t*-test was used to determine statistical significance between datasets, with *p* < 0.05 considered significant.

## 3 | Results

### 3.1 | Phantom Study

**FIGURE 1 |:**
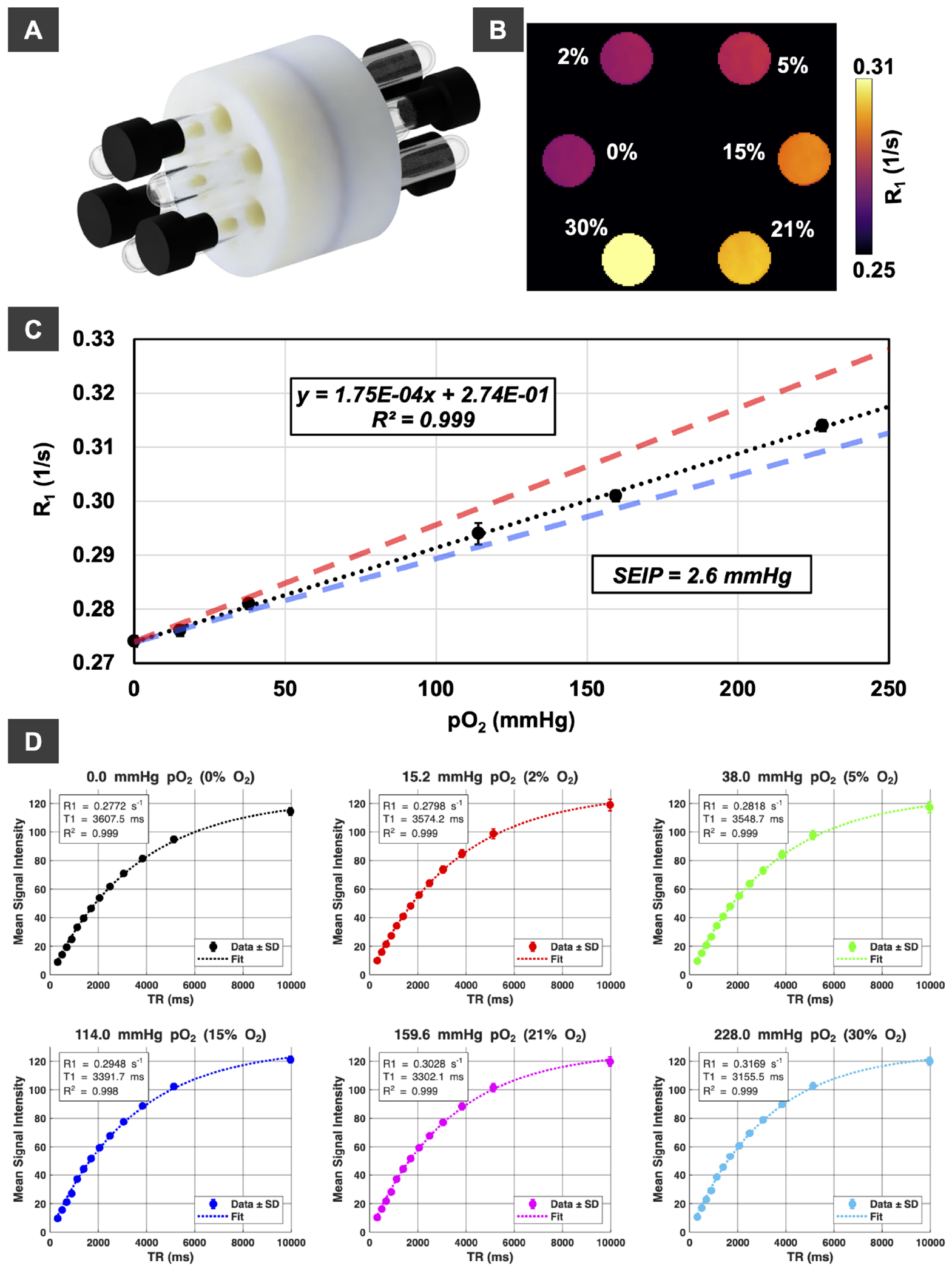
OE-MRI with phantoms. (A) Six deionized water phantoms equilibrated with various concentrations of oxygen. The water is in 5 mm outer-diameter NMR tubes, capped, and sealed with parafilm wax film. (B) R_1_ map for each phantom (0%, 2%, 5%, 15%, 21%, and 30% O_2_) with a resolution of 0.117 × 0.117 mm^2^ (8.53 px/mm). (C) Linear relationship between R_1_ and pO_2_. Slope of the curve (1.75 × 10^−4^ 1/s/mmHg, *R*^2^ = 0.999, *p* < 0.001) matches the expected range for 9.4T(1.42–2.08× 10^−4^ 1/s/mmHg) based on empirical modeling [50]. Blue line represents an approximation of the relaxivity (slope) at 21°C, and the red line is at 40°C. The standard error of inverse prediction (SEIP), or the uncertainty in pO_2_ for a given R_1_,was calculated to be 2.6 mmHg. (D) Results of the saturation recovery T_1_ mapping sequence for each vial.

The phantom experiment established the predictive accuracy of the R_1_ –pO_2_ relationship using saturation-recovery T_1_ mapping and showed that the measured relaxivity matched previously published experimental data [50]. Figure 1A shows a rendering of the phantom setup with the water phantoms symmetrically placed in a 3D-printed holder. The R_1_ map for each phantom (Figure 1B) shows the rate of the spin – lattice relaxation increasing with increasing pO_2_. Figure 1C illustrates the linear correlation between R_1_ and pO_2_ . The slope of the linear fit (or relaxivity) was determined to be 1.75 × 10^−4^ 1/s/mmHg (*R*^2^ = 0.999, *p* < 0.001), which matches well within the expected range (1.42 – 2.08 × 10^−4^ 1/s/mmHg; extrapolated to 9.4T from the empirical model of Bluemke et al., which extends to 8.45 T) [50]. The standard error of inverse prediction (SEIP) was used to estimate the inverse predictive precision of a value on the *y*-axis relative to a value on the *x*-axis based on the root-mean-square error and the slope of the linear curve [51]. The result of this experiment shows how pO_2_ affects the longitudinal relaxation rate (Figure 1D) and that for a given value of R_1_, the pO_2_ value can be determined with an error of 2.6 mmHg.

### 3.2 | OxyChip With GNP Characterization

#### 3.2.1 | Morphology and Composition

**FIGURE 2 |:**
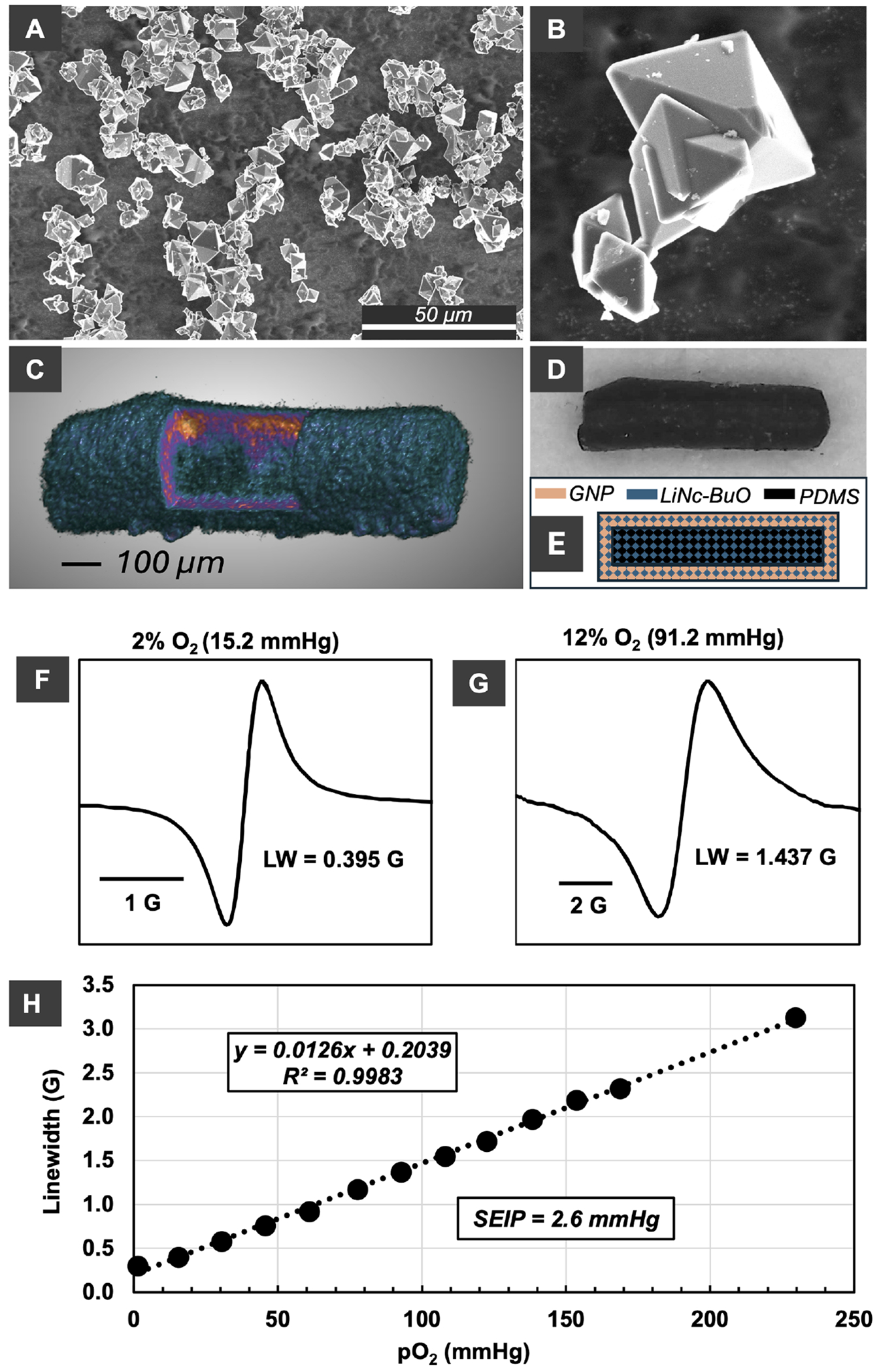
OxyChip with GNP. (A) Scanning electron microscope image of the LiNc-BuO crystals, the EPR oxygen sensing material. (B) Zoomed-in view of a small LiNc-BuO crystal cluster, showcasing the overlapping octahedral structure. (C) High-resolution micro-CT rendering of a completed OxyChip (LiNc-BuO embedded in oxygen-permeable polydimethylsiloxane (PDMS) and coated with gold nanoparticles, GNPs). The bright orange sections of the OxyChip shown in the cutout highlight the presence of the GNPs on the surface of the material. (D) Photograph of an OxyChip (1 mm length, 0.2 mm diameter) through a digital microscope. (E) Cartoon drawing of the OxyChip showing how the LiNc-BuO crystals are embedded entirely in the PDMS polymer with the GNPs deposited on the surface. (F) Continuous wave L-band EPR spectrum of the OxyChip at 2% O_2_ (15.2 mmHg) with a peak-to-peak linewidth (LW) of 0.395 G. (G) EPR spectrum of the same OxyChip at 12% O_2_ (91.2 mmHg) with a peak-to-peak linewidth (LW) of 1.437 G. (H) OxyChip calibration curve. The slope is 0.0126 G/mmHg, with an anoxic linewidth of 204 mG and SEIP = 2.6 mmHg. pO_2_ values were set using controlled gas mixtures verified by an in-line optical oxygen sensor.

Scanning electron microscopy showed LiNc-BuO crystals with characteristic octahedral geometry and dimensions between 4 and 11 μm wide (Figure 2A,B). Micro-CT reconstruction of the GNP-coated OxyChips (Figure 2C) showed the preferential GNP deposition near the surface of the OxyChip, with the heatmap overlay indicating GNP concentration at the brightest points. Visual microscopy (Figure 2D) revealed a smooth OxyChip with no breaks or tears (1 mm length, 250 μm diameter), suitable for implantation. The overall composite structure (Figure 2E) consisted of an oxygen-permeable PDMS matrix containing dispersed LiNc-BuO crystals, coated with GNPs.

#### 3.2.2 | EPR Oxygen Sensitivity

The EPR spectra of the OxyChip demonstrated the oxygen-dependent line broadening (Figure 2F,G). At 15.2 mmHg (2% O_2_), the measured linewidth was 0.395 ± 0.002 G, while at 91.2 mmHg (12% O_2_), the linewidth increased to 1.437 ± 0.002 G, indicative of the interaction between diatomic oxygen and the LiNc-BuO probe. Calibration beyond the physiological range (0–228.0 mmHg, 0%–30% O_2_) in 15.2 mmHg (2% O_2_) increments yielded exceptional linearity (Figure 2H): linewidth (G) = 0.0126 × pO_2_ (mmHg) + 0.2039 (*R*^2^ = 0.9983), corresponding to an oxygen sensitivity of 12.6 mG/mmHg with an anoxic linewidth of 204 mG. The SEIP was 2.6 mmHg, equivalent to the precision achieved in the phantom validation experiments.

### 3.3 | *In Vivo* qOE-MRI Implementation

#### 3.3.1 | OxyChip Implantation and Localization

**FIGURE 3 |:**
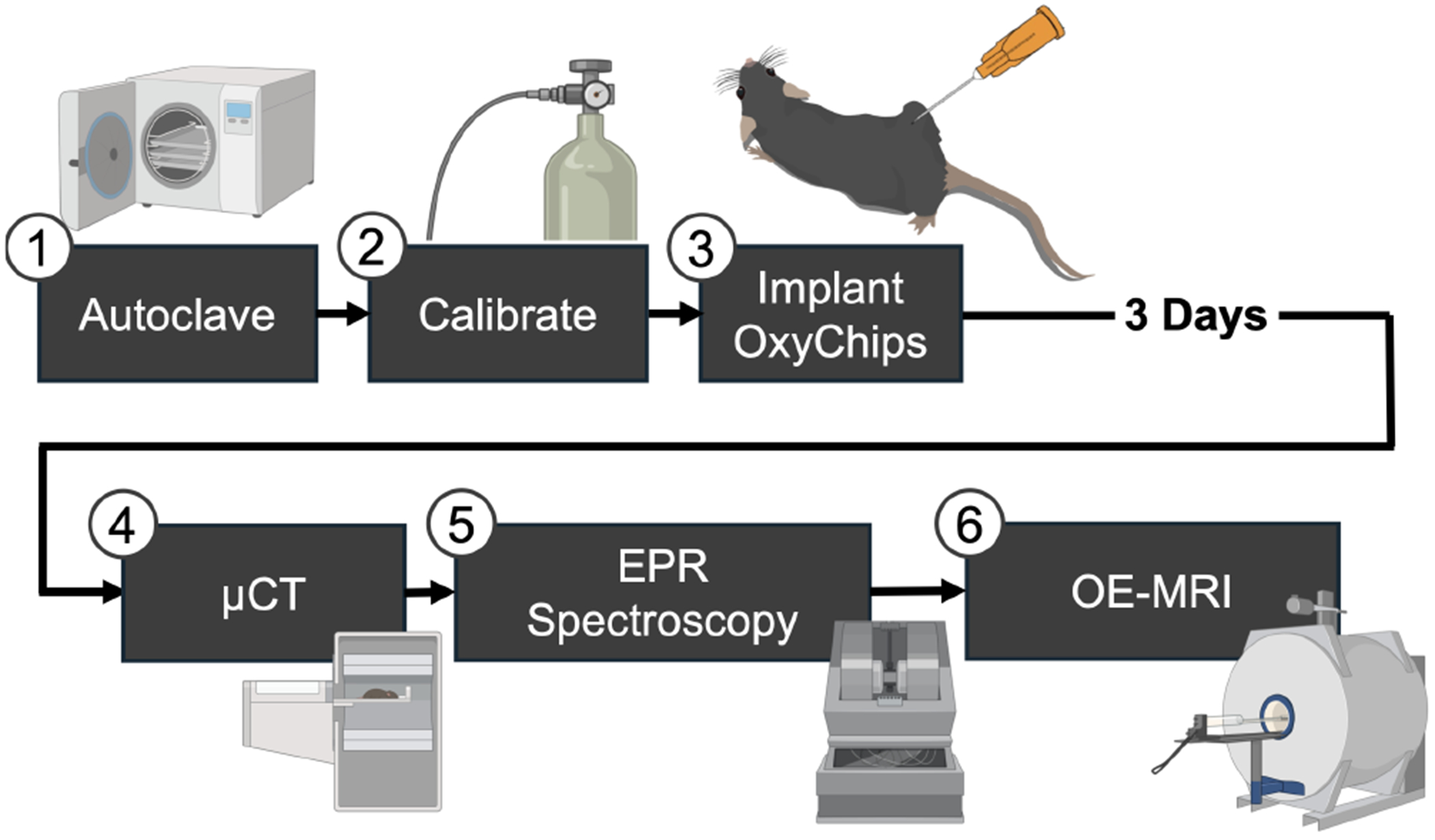
Experimental procedure. Steps of the procedure. After the OxyChips are prepared and soaked in a GNP solution for 24-h, they are placed in a 23Ga syringe needle and stored in a self-sealing sterilization pouch along with a stylet. (1) The OxyChips and needles are then autoclaved for 30 min. (2) One OxyChip is removed from the batch and used for calibration. Calibration involves using a series of mixed gas concentrations from 0% to 30% O_2_ (verified with an optical oxygen gas sensor) to measure the EPR linewidth at each gas concentration and to determine the relationship between EPR linewidth and pO_2_. (3) Three OxyChips are then implanted into the tumor located on the right flank of the mouse. A control injection (no OxyChip) was also performed to verify tissue recovery later. A 3-day recovery period was observed following implantation to allow tissue stabilization. (4) The tumor is imaged using a micro-CT scanner to determine the exact positions of the OxyChips in the tissue. (5) The mouse is placed in a continuous wave L-band EPR spectrometer while breathing 21% O_2_ and 1.5% isoflurane (induced with 3% isoflurane) and kept warm under a heat lamp. The EPR spectrum of each OxyChip is collected. The breathing gas is then swapped to 100% O_2_, and the measurements are repeated. (6) Following a small 2-h waiting period to allow for the mouse to recover from anesthesia and to re-hydrate, it is transferred to the 9.4 T pre-clinical MRI system. The mouse is placed in a custom-made volume coil while breathing 21% O_2_ and 1.5% isoflurane (induced with 3% isoflurane) and kept warm using forced air. A series of saturation-recovery relaxometry sequences is performed to map tissue T_1_ while the mouse breathes 21% O_2_, then 100% O_2_.

**FIGURE 4 |:**
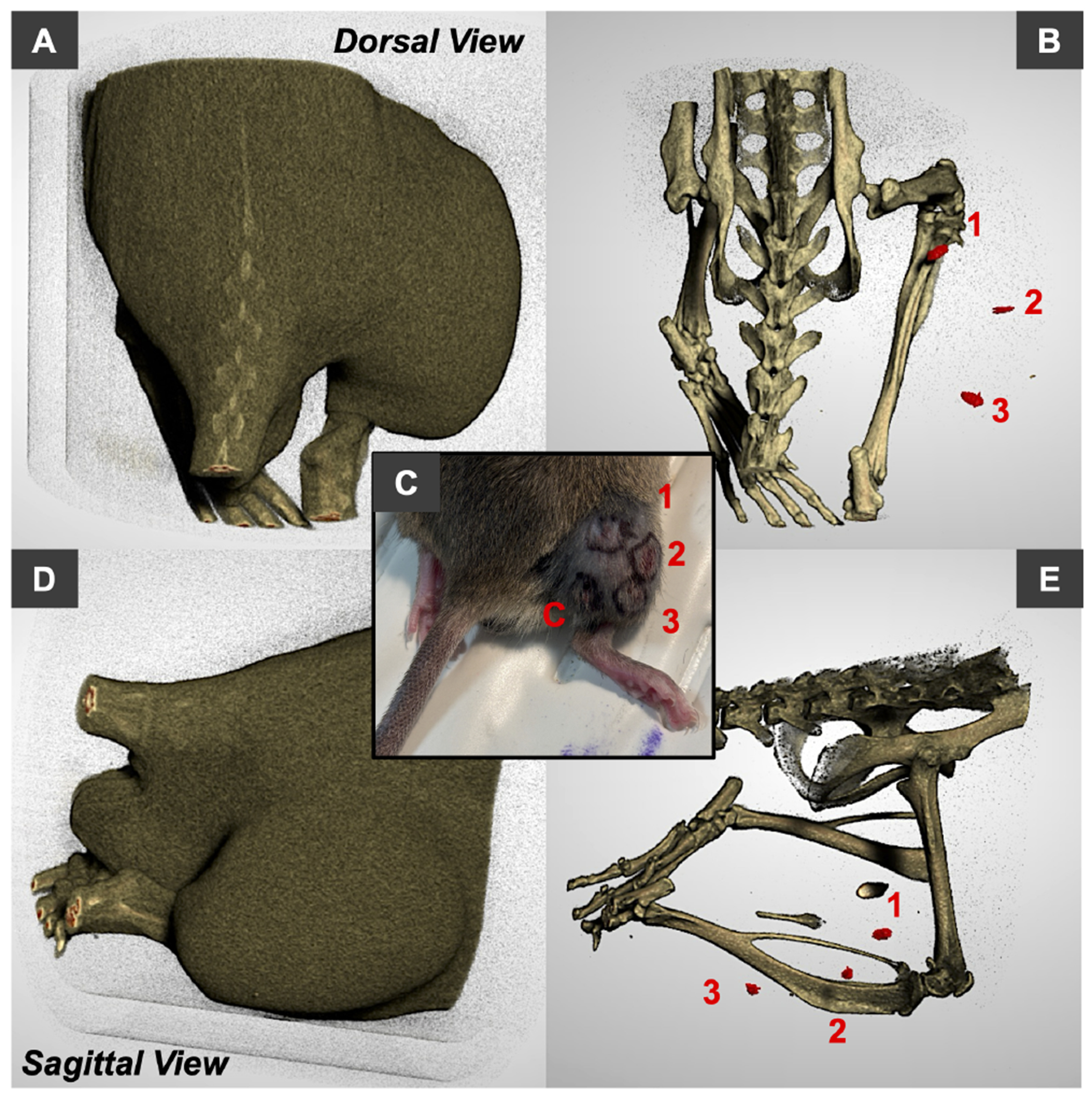
Implanted OxyChips in a mouse. (A) 3D reconstruction of the mouse from the sacral region to the leg region (dorsal view). (B) Dorsal view of the mouse skeleton and the relative positions of each OxyChip (1–3) visible due to the GNPs. (C) Photographic image showing the implantation sites of the OxyChips and the control injection 3 days prior. (D) 3D reconstruction of mouse tissue (sagittal view). (E) Sagittal view of the mouse skeleton and the relative position of each OxyChip (1 – 3).

The complete qOE-MRI workflow (Figure 3) was implemented as a proof-of-concept in SCC7 tumor-bearing mice; full results are reported for one animal implanted with three OxyChips, which provided the highest calibration point count and the smallest OxyChip size. Supporting data from a second animal, acquired with a surface coil and two larger OxyChips, are provided in the Supporting Information. After autoclave sterilization, EPR calibration, and implantation of three OxyChips, a 3-day recovery period allowed for tissue stabilization before imaging. Micro-CT provided a high-resolution visualization of OxyChip positions within the tumor. 3D reconstruction from the dorsal perspective (Figure 4A,B) shows the relative positions of the three OxyChips labeled “1”, “2”, and “3” (anterior to posterior), plus a control injection site “C” without an OxyChip. The implantation sites, 3 days before imaging, are shown in Figure 4C, along with the spacing between them. A sagittal view (Figure 4D,E) illustrated another perspective on the spatial distribution of the OxyChips. Tumor volume was approximately 1400 mm^3^ at the time of imaging studies, and growth rate was about 100mm^3^/day.

#### 3.3.2 | EPR Oximetry Measurements

**FIGURE 5 |:**
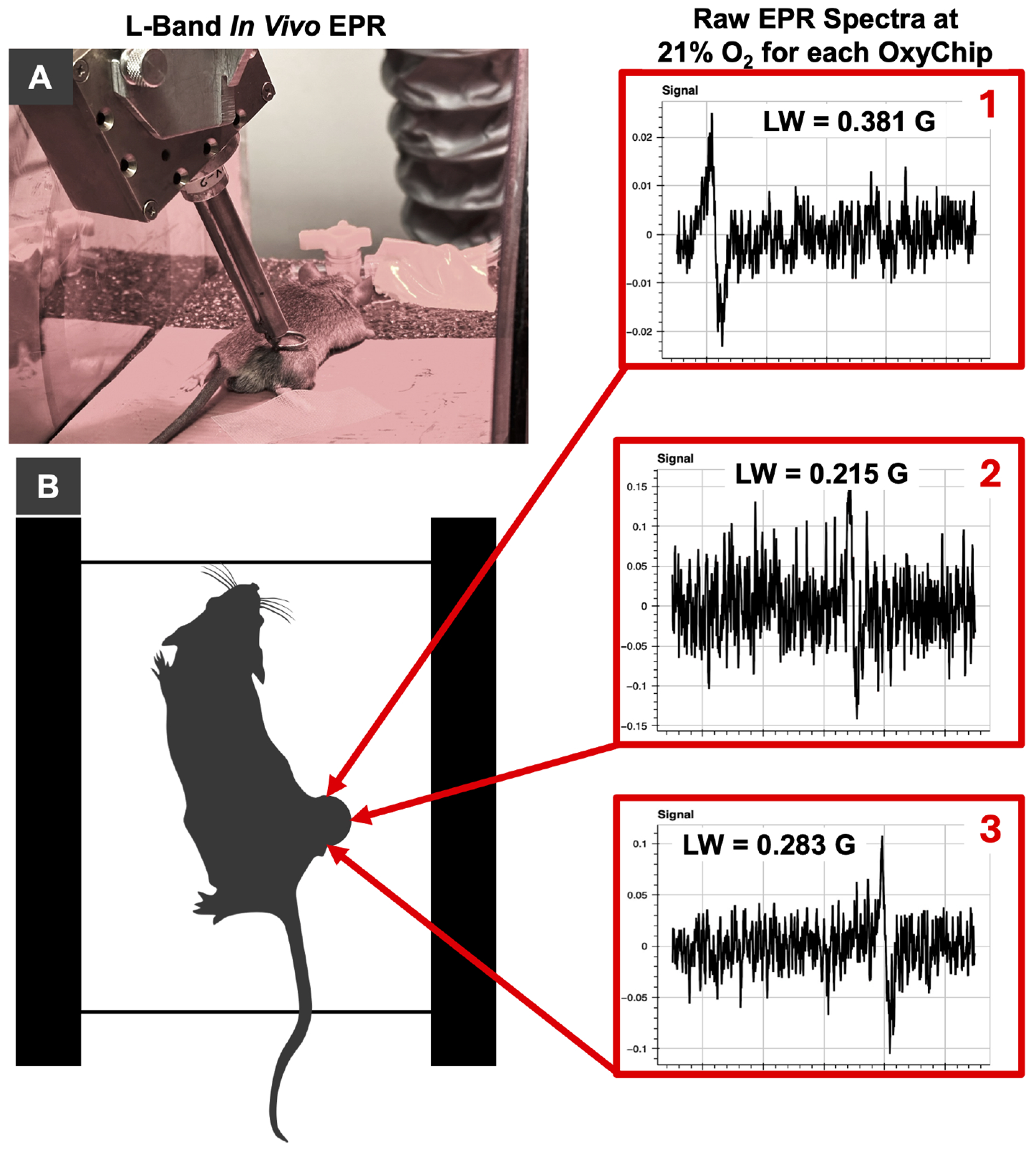
EPR oximetry. (A) Experimental setup of a mouse in a continuous wave EPR spectrometer with a surface resonator placed above the tumor. (B) Cartoon representation of the mouse in the spectrometer (top-down view) and the raw (not denoised) EPR spectra of each OxyChip while the mouse is breathing 21% O_2_ . The measured linewidths are 0.381, 0.215, and 0.283 G, respectively, for OxyChips 1, 2, and 3.

L-band EPR spectroscopy was then performed with the animals positioned at the magnet isocenter (Figure 5A). Using a surface resonator, the EPR spectra were acquired from each implanted OxyChip. Under normoxic breathing conditions (21% O_2_), representative spectra for each OxyChip (Figure 5B) revealed marked heterogeneity in tumor tissue oxygenation. Measured linewidths were 0.381 ± 0.002 G (OxyChip 1), 0.215 ± 0.002 G (OxyChip 2), and 0.283 ± 0.002 G (OxyChip 3), corresponding to pO_2_ values of 14.3 ± 2.6, 1.2±2.6, and 6.6 ± 2.6 mmHg, respectively. OxyChip 2, positioned in the tumor core, exhibited severe hypoxia under normal air-breathing conditions.

Following a 20-min hyperoxic challenge (100% O_2_), EPR measurements were repeated at identical positions. Linewidths changed to 0.401 ± 0.002 G, 0.293 ± 0.002 G, and 0.254 ± 0.002 G for OxyChips 1, 2, and 3, corresponding to pO_2_ values of 16.0 ± 2.6, 6.5 ± 2.6, and 4.3 ± 2.6 mmHg. The OxyChip at the muscle-adjacent position (OxyChip 1) exhibited pO_2_ above the 10-mmHg threshold and showed a moderate response to the hyperoxic challenge (+1.6 mmHg). In comparison, the severely hypoxic core (OxyChip 2) demonstrated more improvement (+5.3 mmHg), and the peripherally located OxyChip (OxyChip 3) exhibited a decrease in tumor oxygenation (−2.3 mmHg). These point measurements, however, underscore the need for volumetric pO_2_ visualization.

#### 3.3.3 | OE-MRI Acquisition

**FIGURE 6 |:**
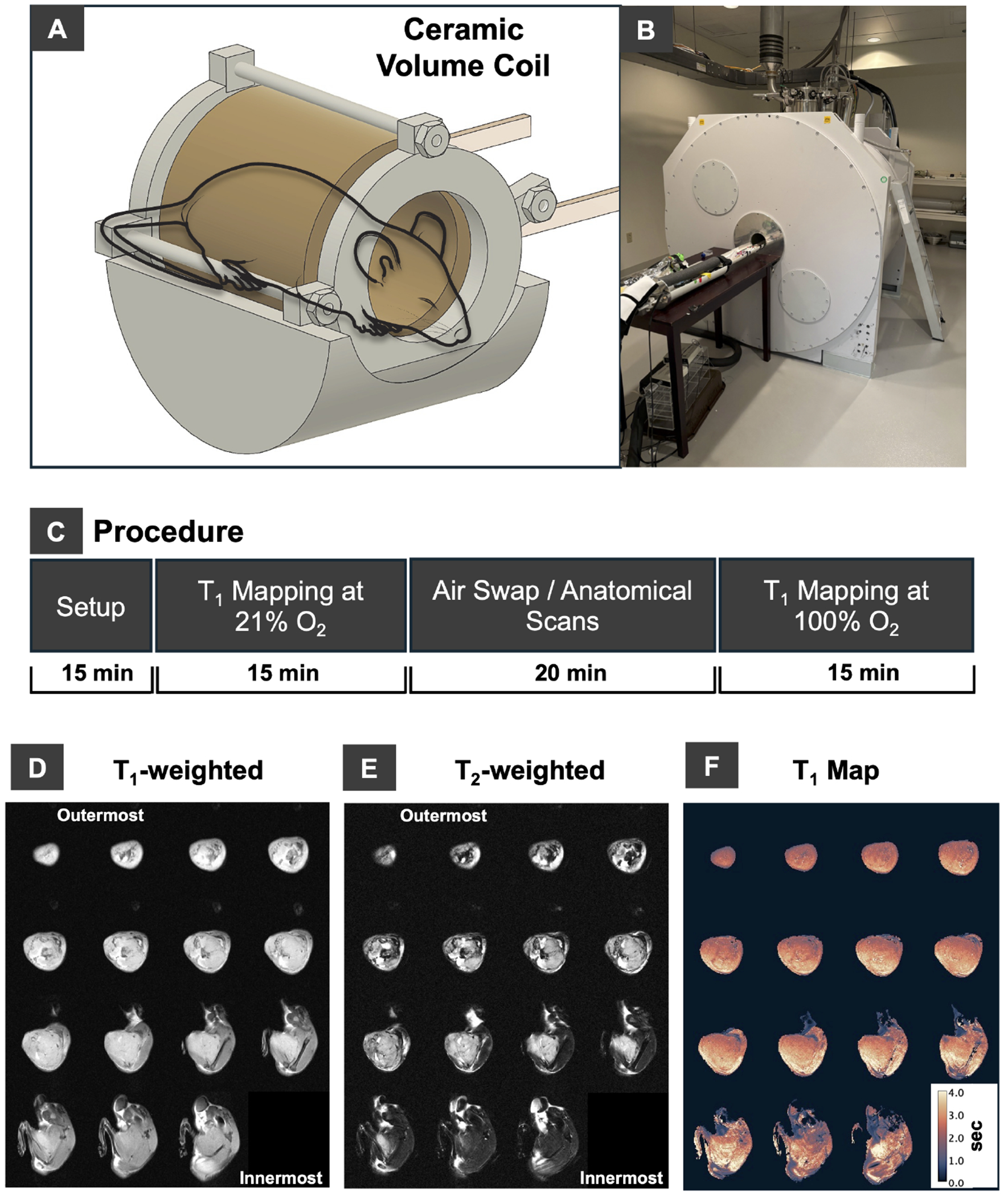
OE-MRI setup. (A) Cartoon representation of the mouse in the custom-made ceramic dielectric volume coil. (B) 9.4T pre-clinical MR imaging system. (C) OE-MRI procedure. (D) T_1_-weighted anatomical scan of the tumor and leg tissue. (E) T_2_ -weighted anatomical scan of the tumor and leg tissue. (F) T_1_ map of the tumor and leg tissue while breathing 21% O_2_ .

Animals were transferred to a 9.4 T pre-clinical MRI scanner and placed in a custom-built ceramic volume coil (Figure 6A,B) [45]. The imaging protocol (Figure 6C) replicated the breathing conditions during the EPR oximetry measurements. Multi-slice T_1_-weighted (Figure 6D) and intermediate T_2_-weighted (Figure 6E) anatomical images provided a detailed view of the tumor architecture from the outermost periphery (top-left) through to the innermost muscle-adjacent tissue (bottom-right). At the time of imaging, no discernible tissue damage was observed at either implantation site, indicating successful tissue recovery following OxyChip and control injections. GNP-induced susceptibility artifacts in the phase-encoding direction simplified OxyChip localization within the tumor. The measured spin – lattice relaxation time map at 21% O_2_ (Figure 6F) depicts the results of the relaxometry protocol and reveals spatial variation in baseline tissue relaxation properties [52]. The RARE readout using multiple echoes introduces moderate T_2_ weighting into the T_1_ estimate, resulting in an effective T_1_ measurement. Sub-ROIs of tissue nearest to the OxyChip location (less than 300 μm from the OxyChip) yielded mean measured R_1_ = (1/T_1_) values under normoxic conditions of 0.425 ± 0.1 s^−1^ (OxyChip 1), 0.323 ± 0.05 s^−1^ (OxyChip 2), and 0.347 ± 0.04 s^−1^ (OxyChip 3). Under hyperoxic conditions, these values changed to 0.443 ± 0.1, 0.363 ± 0.08, and 0.330 ± 0.02 s^−1^, respectively, demonstrating the effects of oxygen-dependent relaxation.

#### 3.3.4. | Tissue-Specific Calibration and pO_2_ Mapping

**FIGURE 7 |:**
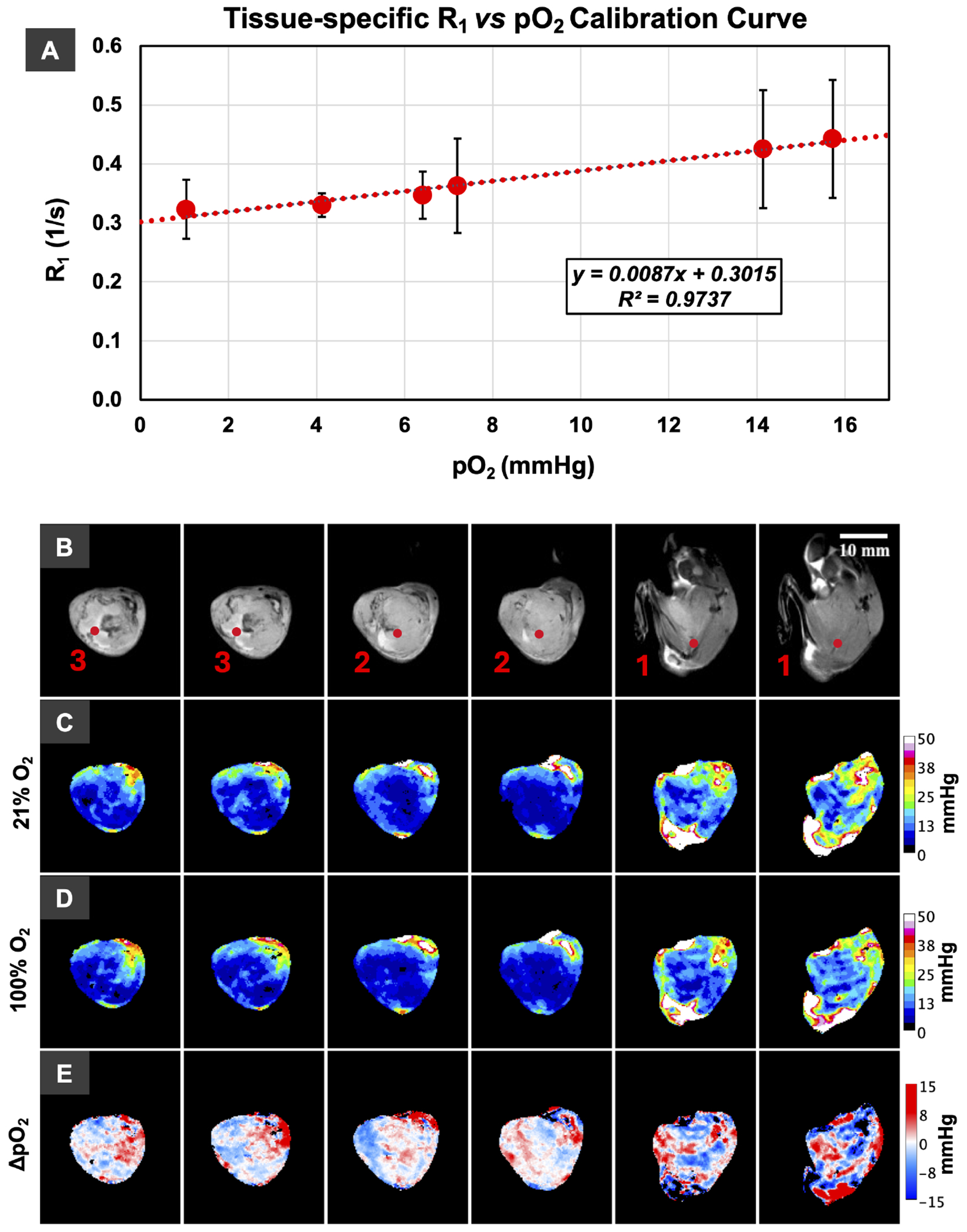
qOE-MRI results. (A) Tissue-specific R_1_ –pO_2_ linear calibration curve. (B) T_1_-weighted anatomical scan of the tumor and surrounding leg muscle showing the slices where the OxyChips are visible due to the slight susceptibility artifacts caused by the GNPs. Each OxyChip is demonstrated in two slices — same OxyChip labeling convention as in Figures 4 and 5. An R_1_ map is generated by taking the inverse of the T_1_map. The average R_1_ value of the tissue nearest to the OxyChip (within 300 μm) is recorded and averaged between slices. A calibration curve is generated by correlating the recorded R_1_ values and the measured pO_2_ values from EPR oximetry for each breathing condition. (C) pO_2_ map of the tumor and surrounding leg muscle at 21% O_2_. (D) pO_2_ map of the tumor and surrounding leg muscle at 100% O_2_. (E) Delta pO_2_ between 100% and 21% O_2_ breathing conditions. Red-colored numbers indicate OxyChip positions.

Combining the results of the pO_2_ measurements from the EPR oximetry and the R_1_ measurements from OE-MRI, a correlation curve can be generated (Figure 7A). A linear function is fitted to set a calibration curve from R_1_ maps to pO_2_ maps: R_1_ (s^−1^)=0.0087 • pO_2_ (mmHg) + 0.3015 (*R*^2^ = 0.9737), where 0.0087 (s•mmHg)^−1^ represents the effective oxygen-dependent relaxivity coefficient and 0.3015 s^−1^ represents the baseline relaxation rate at anoxia. This calibration procedure enables the conversion of whole-tumor R_1_ maps to spatially defined pO_2_ maps. Multi-slice T_1_-weighted anatomical images (Figure 7B) identified slices containing each OxyChip (labeled “1”, “2”, and “3”). Application of the calibration equation generated absolute pO_2_ maps under normoxic (Figure 7C) and hyperoxic (Figure 7D) breathing conditions. Quantitative pO_2_ maps revealed substantial spatial variation, with hypoxic regions (blue, < 10 mmHg) predominantly in the tumor core and better-oxygenated regions (green to red, > 20 mmHg) near the periphery and muscle-tumor interface. Difference mapping (ΔpO_2_, Figure 7E) highlighted oxygen-responsive regions (red) and areas with minimal (white) or even negative responses (blue), indicating complex vascular reactivity patterns.

#### 3.3.5. | Spatial Analysis of Tumor Oxygenation

**FIGURE 8 |:**
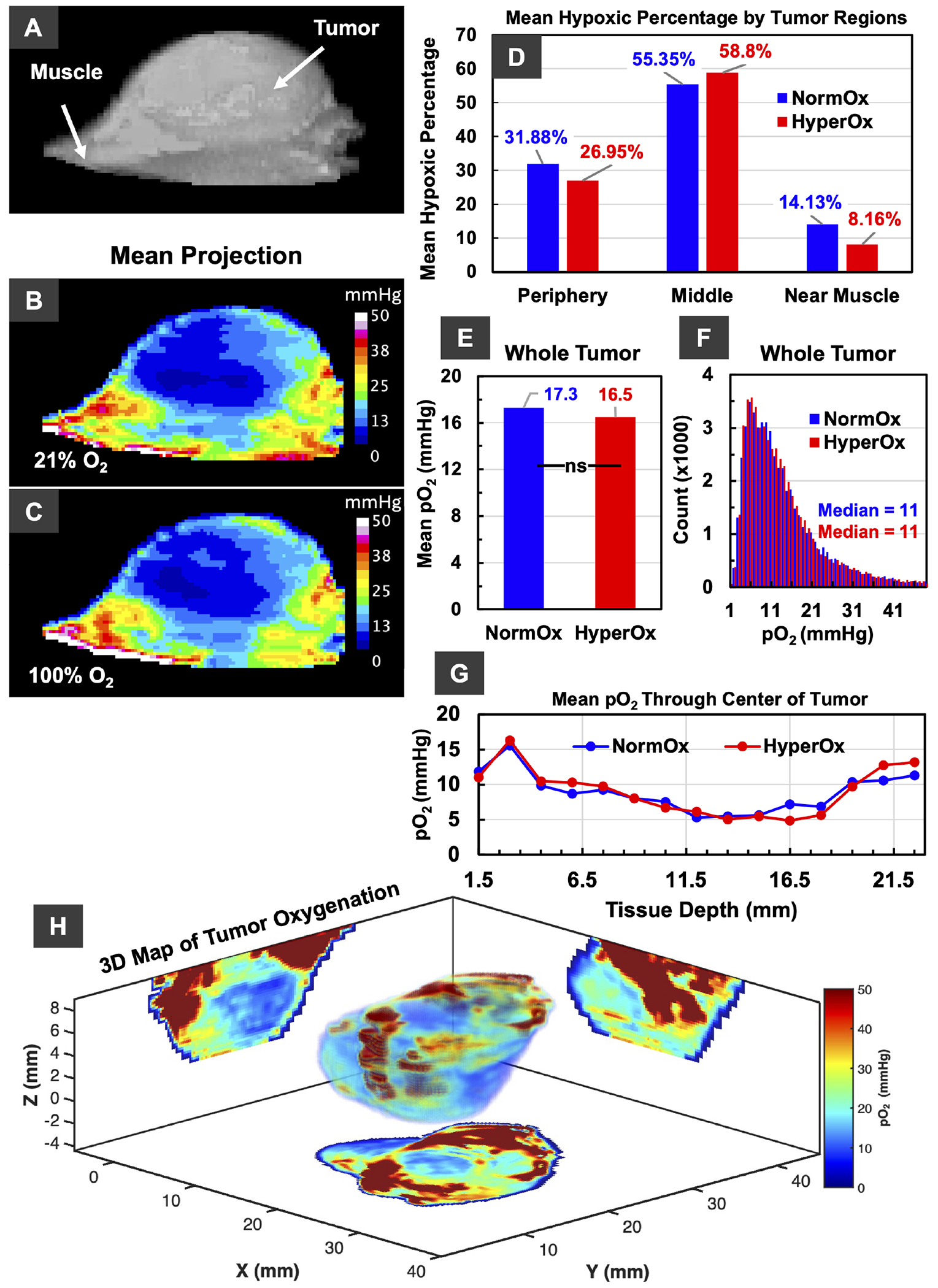
3D rendering of tumor pO_2_.(A)T_1_-weighted projection showing the relative size and position of the tumor to the muscle tissue for reference. (B) Average pO_2_ projection map at 21% O_2_. (C) Average pO_2_ projection map at 100% O_2_ showing areas of shifting oxygenation compared to B. (D) Mean change in the percentage of tissue that is hypoxic by tumor region. The tumor “periphery” represents the regions near the outermost section of the tumor, furthest from the body. The “middle” region is represented in the inner core of the tumor. The “near muscle” region represents the innermost tissue nearest to the body. The inner and outermost areas respond well to the oxygen challenge, showing a decrease in hypoxic tissue. Still, the extremely hypoxic core does not show a reduction in the hypoxic fraction. (E) The average pO_2_ across the entire tumor shows no significant changes after the oxygen challenge. (F) Histograms of pO_2_ for each breathing condition across the tumor as a whole show no significant changes in tissue oxygenation. (G)A 5 × 5mm^2^ track from the outermost tumor region to the innermost, showing how the average pO_2_ changes through the center of the tumor for each breathing condition. (H) 3D map of tumor oxygenation with averaged projections along the principal axes.

A 3D reconstructed projection of the 2D T_1_-weighted images (Figure 8A) provides a visualization of tumor morphology and the surrounding muscle tissue for reference. Corresponding 3D mean pO_2_ projections under normoxic (Figure 8B) and hyperoxic (Figure 8C) conditions revealed the extent of hypoxia throughout the tumor volume. A regional analysis (Figure 8D) quantified the hypoxic fraction (< 10 mmHg) across three anatomically distinct locations. Under normoxic breathing, the tumor periphery exhibited moderate hypoxia (31.88% hypoxic), the core showed severe hypoxia (55.35% hypoxic), while the muscle-adjacent region demonstrated relative normoxia (14.13% hypoxic). Hyperoxic breathing produced a differential regional response: the tumor periphery showed some improvement (26.95% hypoxic, Δ = −4.93%), the core remained severely hypoxic (58.8% hypoxic, Δ = +3.45%), while the muscle-adjacent tissue exhibited substantial improvement (8.16% hypoxic, Δ = −5.97%). These data indicate limited oxygen diffusion to the tumor core, while peripheral tissue sees some benefit of hyperoxygen breathing. Compared to the EPR measurement at the center of the tumor, the pO_2_ map showed that despite a measurable increase at a small point in the core as reported by the OxyChip, the average core pO_2_ did not increase.

Whole-tumor histograms (Figure 8E,F) demonstrated no significant change in mean pO_2_ between breathing conditions (17.3 mmHg vs. 16.5 mmHg for normoxic and hyperoxic conditions, respectively, *n* = 15, *p* = 0.179, Cohen’s *d* = 0.37). Both conditions exhibited identical median pO_2_ values of 11 mmHg, with minor variations. The depth-dependent profile through a 5 × 5 mm^2^ central tumor core spanning from the superficial region (1.5 mm depth) through to the deep muscle-tumor interface (22.5 mm) revealed characteristic spatial pO_2_ gradients (Figure 8G). At the tumor’s outer edge, tissue oxygenation was higher, though the oxygen challenge response was minimal. Moving inward toward the center of the tumor (9 – 16.5 mm), the tumor tissue is hypoxic and unresponsive to the hyperoxic breathing challenge. In contrast, for tissue closer to the muscle-tumor interface (21 – 22.5 mm), there is a positive response to the oxygen challenge. The persistent hypoxic core despite hyperoxic breathing suggests diffusion-limited oxygen delivery, which is consistent with poorly vascularized tumor regions.

## 4 | Discussion

This study presents the first application of combining EPR oximetry with OxyChip and 9.4 T OE-MRI to generate high-resolution pO_2_ maps of tumor tissue. Each modality offers distinct advantages: EPR spectroscopy oximetry provides accurate, discrete measurements of pO_2_, while OE-MRI generates spatially resolved maps of tissue response to changes in oxygenation. Individually, each technique provides a partial view; their combination enables comprehensive spatial assessment of tumor oxygenation.

The qOE-MRI method demonstrates clinically relevant translational potential. The protocol requires OxyChip implants, a clinical EPR spectrometer, and an MRI scanner. The spatial resolution of qOE-MRI depends on the MRI scanner’s field strength and signal-to-noise ratio. In this study, a 9.4 T MRI was employed to achieve high resolution; thus, higher-field scanners would further improve pO_2_ mapping accuracy. Compared with other hypoxia-imaging approaches — including FMISO-PET, BOLD-MRI, TOLD-MRI alone, and DCE-MRI — qOE-MRI offers non-radioactive, repeatable tissue oxygen measurements with high spatial resolution (limited by the MR scanner) and is compatible with MR-LINAC platforms now in clinical use. The change in R_1_ under an oxygen challenge has also been proposed as a predictive marker of radiation response [53–56]. However, the radiobiological relevance of oxygenation changes is threshold-dependent; that is, the oxygen enhancement ratio is primarily meaningful when hypoxic regions (pO_2_ < 10 mmHg) transition above the radiosensitization threshold (∼20 mmHg). Relative ΔR_1_ alone cannot confirm whether this transition has occurred, emphasizing the value of absolute calibration to build radiobiologically interpretable maps.

Phantom validation of the T_1_ saturation recovery technique demonstrated excellent linearity of the R_1_–pO_2_ relationship (*R*^2^ = 0.999), confirming the linear oxygen-dependent correlation of the spin–lattice relaxation rate and establishing the accuracy of the saturation recovery relaxometry method (2.6 mmHg). Ideally, ground-glass stoppered tubes would have been used to provide a more hermetic seal, but oxygen diffusion is slow and was minimized by imaging the samples quickly after sealing the tubes. Characterization of the OxyChip confirmed its suitability for accurate EPR measurements, given its small size (1 mm length×250 μm diameter) and visibility across multiple imaging modalities. The GNP coating enabled localization via MRI without affecting EPR sensitivity (12.6 mG/mmHg) and facilitated identification of the OxyChips with micro-CT. In this study, the SEIP for the OxyChip was 2.6 mmHg, but for larger OxyChips, which provide better SNR and better resonators, the SEIP can be as low as 0.3 mmHg [34].

The results of the pO_2_ mapping highlight the extensive hypoxia in the SCC7 tumor, which is well known to be extremely hypoxic and therefore radioresistant [57–59]. SCC7 tumors, known for their limited oxygen responsiveness, nonetheless provided an informative test case for evaluating the qOE-MRI protocol. The hypoxic fraction in these tumors varies widely across studies (approximately 10% – 80%), and the regional analysis in Figure 8D demonstrates that sub-volumes near the tumor periphery and muscle interface retain oxygen responsiveness even when whole-tumor mean pO_2_ does not change significantly [60]. This regional heterogeneity—invisible to a single-point EPR measurement—has direct implications for hypoxia-directed dose escalation. The pO_2_ maps show significant spatial differences throughout the tumor microenvironment. It was found that the periphery and the muscle-adjacent tissue respond much more profoundly to oxygen challenges, whereas the hypoxic center showed little response. This result is consistent with the established hypoxic core phenotype of SCC7 tumors at large volumes, where prior EPRI studies have documented progressive hypoxic expansion and reduced micro-vessel density with increasing tumor size. Meanwhile, tissue near better-vascularized normoxic tissue has a better chance of undergoing changes during an oxygen challenge [6, 10, 19, 57]. We note that not all solid tumors exhibit this spatial distribution. Future studies will include tumor models with stronger global oxygen responsiveness to further evaluate qOE-MRI sensitivity across the full range of tumor phenotypes.

While EPR measurements at the OxyChip locations provide direct pO_2_ quantification with good precision (SEIP = 2.6 mmHg), the extrapolation to surrounding tissue relies on the assumption of a linear R_1_–pO_2_ relationship. Non-oxygen-dependent relaxation mechanisms including macromolecular interactions, pH variations, and compartmental water exchange may introduce departures from linearity that diminish the accuracy at regions distant from the calibration points. The limited number of calibration points (*n* = 3) in this proof-of-concept study restricts our ability to detect and correct for such irregularities. Although the hyperoxic challenge aims to isolate an oxygen-only-dependent change, it might not fully control for unknown variables. Future validation by other oxygen-imaging techniques, such as EPR oxygen imaging (EPROI), which provides spatially resolved pO_2_ maps, will help establish the quantitative reliability of qOE-MRI-derived pO_2_ distributions.

The generalizability of tissue-specific calibration curves requires systematic evaluation. The observed R_1_–pO_2_ relationship may vary between tumor types, subjects, and surrounding tissue due to differences in tissue composition, cellularity, necrosis, and edema. This is particularly important as this method takes advantage of both T_1_ and T_2_ oxygen-dependent mechanisms [61]. The RARE multi-echo readout introduces moderate T_2_-weighting into the T_1_ estimate, yielding an effective T_1_; because T_2_ also shortens oxygen-dependently via deoxyhemoglobin susceptibility contributions, the effective relaxivity slope from this sequence may exceed that of a pure spin–lattice measurement [62].

Whether the slope of the R_1_–pO_2_ relationship is tissue-independent also remains an open question. Bluemke et al. demonstrated using a three-compartment model that effective tissue relaxivity departs from the pure aqueous value as a function of blood volume fraction and oxygen extraction fraction, supporting the need for *in situ* calibration rather than a universal constant [63]. The addition of ^19^F MRI or other quantitative oxygen reporters in future studies could provide independent spatial validation of the qOE-MRI-derived pO_2_ distributions. Whether a generalized calibration can be applied across tumor types, or whether subject-specific calibration is essential, requires a larger multi-subject study, which may be a necessary step toward minimizing sensor burden in clinical use.

A few additional points warrant further discussion. Dynamic T_1_ mapping during the oxygen equilibration window would be interesting to explore with qOE-MRI, but was not feasible in this study with the saturation recovery protocol (∼15 min per acquisition at 9.4 T); faster methods such as variable flip angle or Look-Locker acquisitions would enable temporal sampling of the oxygen response, including detection of transient effects. Regarding clinical translation, OxyChip implantation is minimally invasive and has established safety and feasibility in human tumors at depths accessible to current surface resonators; advances in ceramic resonators and needle-based sensors now extend measurable depth beyond 30 mm, substantially broadening the patient population that could benefit [33, 34, 64, 65].

## 5 | Conclusion

This study establishes the first implementation of quantitative oxygen-enhanced MRI (qOE-MRI), combining EPR oximetry with OE-MRI to generate spatially delineated, absolute pO_2_ maps of tumor tissue *in vivo*. The phantom validation confirmed excellent linearity of the R_1_–pO_2_ relationship at 9.4 T. The OxyChip demonstrated reliable EPR oxygen sensitivity and multi-modal visibility facilitated by a GNP coating. *In vivo* experiments in an SCC7 mouse tumor model produced quantitative pO_2_ maps that revealed substantial spatial variation in tumor oxygenation, with the hypoxic core showing limited responsiveness to hyperoxic breathing. Meanwhile, the peripheral and muscle-adjacent tissue exhibited a meaningful oxygen response. These findings are consistent with established models of tumor hypoxia, in which vascular dysfunction and diffusion-limited oxygen transport collectively drive heterogeneous oxygenation across the tumor volume.

The results demonstrate a pilot protocol for qOE-MRI as a potential tool for spatially resolving tumor oxygenation. Future studies should focus on expanding to larger cohorts and cross-validating with independent spatial pO_2_ techniques such as EPROI. The qOE-MRI methodology leverages existing EPR technology alongside standard pre-clinical and clinical imaging equipment, requiring no radioactive contrast agents and enabling repeatable measurements with high spatial resolution. These practical advantages position qOE-MRI as a promising tool for hypoxia-guided radiation therapy planning.

## Supplementary Material

Supplementary Data

Additional supporting information can be found online in the Supporting Information section. Figure S1. Validation of OE-MRI protocol in a mouse brain. (A) PD-w image of the brain. (B) R_1_ map post-nitrogen asphyxiation. (C) R_1_ map while breathing 21% O_2_. (D) R_1_ map while breathing 100% O_2_. (E) Experimental protocol. (F) Histogram of the whole brain for each condition. Figure S2. (A) Upper part of mouse brain with anatomical atlas color-coded. (B) Mean R1 value for the brain for each breathing condition. (C) Mean R1 for each brain region and breathing condition. (D) Table of results from brain regions. Figure S3. T1w images of the OxyChips in tissue. Red arrows identify the OxyChips. Yellow arrows identify the GNP artifacts. Figure S4. (A) micro-CT of OxyChip positions. (B) SEM scan of LiNc-BuO crystal. (C) OxyChip. (D) L-band EPR measurement of OxyChips. (E) OxyChip calibration curve. (F) R1 vs. pO_2_ calibration curve. (G) T1w image of tumor and OxyChip positions. (H) R1 and pO_2_ maps at 21% O_2_. (I) R1 and pO_2_ maps at 100% O_2_.(J) ΔR1 and ΔpO_2_ maps (100% - 21% O_2_). Figure S5. R_1_–pO_2_ calibration curve from five OxyChips from two mice.

## Data Availability

The data that support the findings of this study are available from the corresponding author upon reasonable request.
