# Supplementary material for: Combining Oxygen-Enhanced MRI and Electron Paramagnetic Resonance Oximetry for Quantitative OE-MRI (qOE-MRI) as a Method for Mapping Absolute Oxygen Levels (pO2) in Tumors
Source: Magn Reson Med. Author manuscript; Available in PMC 2026 Jun 17. (PMC13270811; doi:10.1002/mrm.70418)
Supplement: Supplementary Data [file NIHMS2180077-supplement-Supplementary_Data.docx]

# Supplemental Data

**Combining Oxygen-Enhanced MRI and Electron Paramagnetic Resonance Oximetry for Quantitative OE-MRI (qOE-MRI) as a Method for Mapping Absolute Oxygen Levels (pO_2_) in Tumors**

Conner S. Ubert,^1,2^ Victor B. Kassey,^1^ Maciej M. Kmiec,^1^ Diana J. Wallin,^3^ Alireza Kheirollah,^4^ Sergey V. Petryakov,^1^ Ryan C. O’Connell,^1^ Philip E. Schaner,^5^ and Periannan Kuppusamy^1,2,5^

*^1^ Department of Radiology, Geisel School of Medicine, Dartmouth College, Hanover, NH, USA*

*^2^ Thayer School of Engineering, Dartmouth College, Hanover, NH, USA*

*^3^ Department of Psychiatry, Geisel School of Medicine, Dartmouth College, Hanover, NH, USA*

*^4^ Department of Surgery, Geisel School of Medicine, Dartmouth College, Hanover, NH, USA*

*^5^ Department of Radiation Oncology and Applied Sciences, Geisel School of Medicine, Dartmouth College, Hanover, NH, USA*

# 1. OxyChip

## 1.1 Fabrication

OxyChip is an implantable electron paramagnetic resonance (EPR) oxygen sensor capable of accurately measuring tissue pO_2_. OxyChip combines the oxygen-permeable polydimethylsiloxane (PDMS) and the EPR spin probe, lithium octa-n-butoxy-naphthalocyanine (LiNc-BuO) to form a biocompatible material for measuring tissue oxygenation. LiNc-BuO crystals were synthesized in-house using a previously published procedure (Pandian et al. 2003). OxyChips were made from 20% LiNc-BuO:PDMS, which was fabricated by mixing medical-grade platinum silicone elastomer base and catalyst in a 10:1 ratio, then adding LiNc-BuO crystals and platinum crosslinking accelerator. The mixture was degassed under vacuum and drawn into a 0.25-mm inner diameter PTFE tube under negative pressure. After curing at 80ºC for 24 hours, the chip string was drawn from the tubing and cut into 1 mm segments for use.

Gold nanoparticles (GNPs) were incorporated onto the OxyChip surface to enhance image visibility using the method described by Kmeic et al. (2022). The OxyChips were immersed in a solution of gold (III) chloride trihydrate (10% vol) in 200-proof pure ethanol (90% vol) and stirred magnetically at approximately 150 rpm at room temperature for 24 hours. The OxyChips were then rinsed thoroughly with ethanol and dried in room air.

***1.2 Characterization***

The size and morphology of the LiNc-BuO crystals were characterized using a Helios 5 CX DualBeam scanning electron microscope (Thermo Fisher Scientific, Waltham, MA, USA) operating at an accelerating voltage of 5 kV, current of 21 pA, and a working distance of 4.1 mm. Representative images were acquired at 2500x magnification. The visibility of GNP-incorporated OxyChips for localization was evaluated using a Quantum GX3 microCT scanner (Rigaku Corporation, Tokyo, Japan). Individual OxyChips were positioned in a melamine sponge and scanned using the following acquisition parameters: tube voltage = 60 kVp, tube current = 200 µA, exposure time = 4 minutes, field of view = 82 mm, isotropic voxel size = 28.6 µm, matrix size = 2864 × 2864 pixels, slice thickness = 28.6 µm. Images were reconstructed using Dragonfly 3D software (Comet Technologies Canada Inc). Gross morphology and dimensional measurements of complete OxyChips were assessed using an LCD Digital Microscope GOCHIFIX connected via USB to a computer for image capture. The OxyChip was positioned on a white background under the built-in LED illumination and imaged. Length and diameter measurements were performed in ImageJ (NIH, Bethesda, MD) using a calibrated scale derived from the images.

## 1.3 Sterilization

The OxyChips were sterilized prior to calibration and *in vivo* use. Sterilization was performed before calibration because the autoclave process can slightly alter the oxygen sensitivity of the OxyChips. Individual OxyChips were loaded into 23-gauge syringe needles. A stainless-steel stylet was packaged alongside each loaded needle to facilitate OxyChip implantation. The loaded needles and stylets were placed in individual self-sealing sterilization pouches (Crosstex Sure-Check Pouches; Crosstex International Inc., Hauppauge, NY, USA) with integrated steam/ethylene oxide indicator strips. Sterilization was performed using a GETINGE Autoclave Model 533LS Vacuum Steam Sterilizer. The cycle parameters: temperature = 121ºC, chamber pressure = 31.5 PSIA (217 kPa), sterilization time = 60 minutes (exposure phase), followed by a 30-minute drying phase. Post-sterilization, pouches were stored at room temperature.

## 1.4 EPR Measurements

EPR measurements were performed on a custom L-band (~1.2 GHz) spectrometer with a 410-G permanent magnet (Sumitomo Special Metals; Japan), sweep coils (30 G maximum), and 21-kHz field-modulation coils (1.3 G maximum). Field modulation was maintained at approximately 1/3 of the linewidth for all measurements. Sweep time was 6 sec per scan with 50 averages. Spectra were collected with an L-band surface coil resonator.

# 2. Additional *in vivo* OE-MRI Validation

To verify that the variable-TR saturation recovery RARE sequence was appropriate for detecting oxygen-dependent changes in longitudinal relaxation rate *in vivo*, a pilot OE-MRI experiment was performed in a single mouse brain at 9.4T. A C3H mouse was imaged using a 20-mm Bruker surface coil. T_1_ mapping was performed under three breathing conditions: (1) normoxic air (NormOx, 21% O_2_), (2) hyperoxic breathing gas (HyperOx, 100% O_2_), and (3) following nitrogen asphyxiation (NoOx). The NoOx condition was acquired approximately 40 minutes post-sacrifice to allow for complete oxygen consumption by residual tissue (**S.Figure 1**). T_1_ mapping was performed using a variable-TR saturation recovery RARE sequence under each condition with the following parameters: TE = 19.75 ms, TR = [868, 983, 1114, 1265, 1443, 1659, 1935, 2317, 2944, and 5000 ms], ETL = 6, slice thickness = 0.7 mm, slice gap = 0.05 mm, FA = 90°, AVG = 2, FOV = 20 × 20 mm^2^, matrix size = 256 × 192, in-plane resolution = 0.078 × 0.104 mm^2^, BW = 318 Hz/px. R_1_ maps (R_1_ = 1/T_1_) were computed voxel-wise by fitting the saturation recovery signal model to the multi-TR image series.


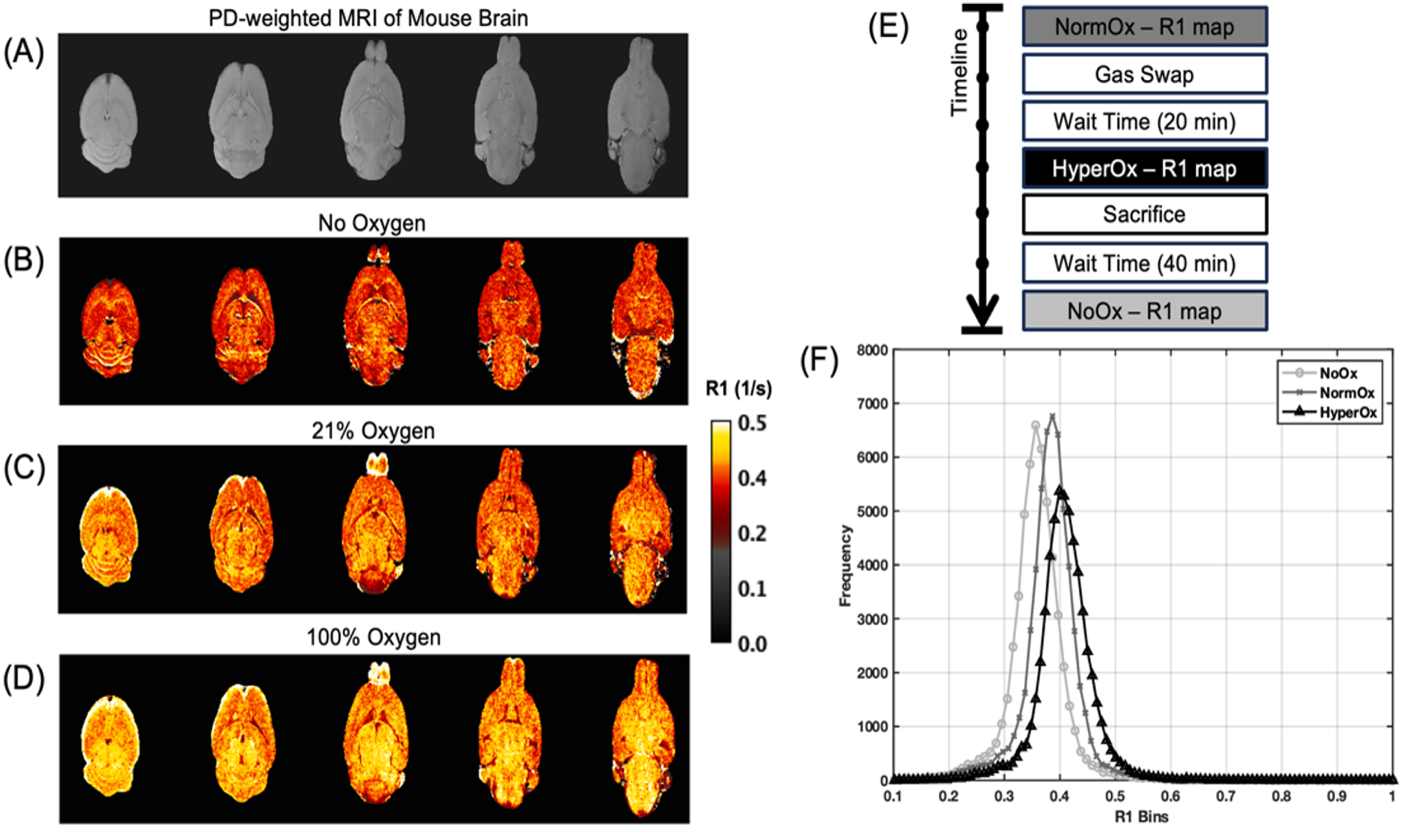


***S.Figure 1:*** *Validation of OE-MRI protocol in a mouse brain. (A) PD-w image of the brain. (B) R_1_ map post-nitrogen asphyxiation. (C) R_1_ map while breathing 21% O_2_. (D) R_1_ map while breathing 100% O_2_. (E) Experimental protocol. (F) Histogram of the whole brain for each condition*.


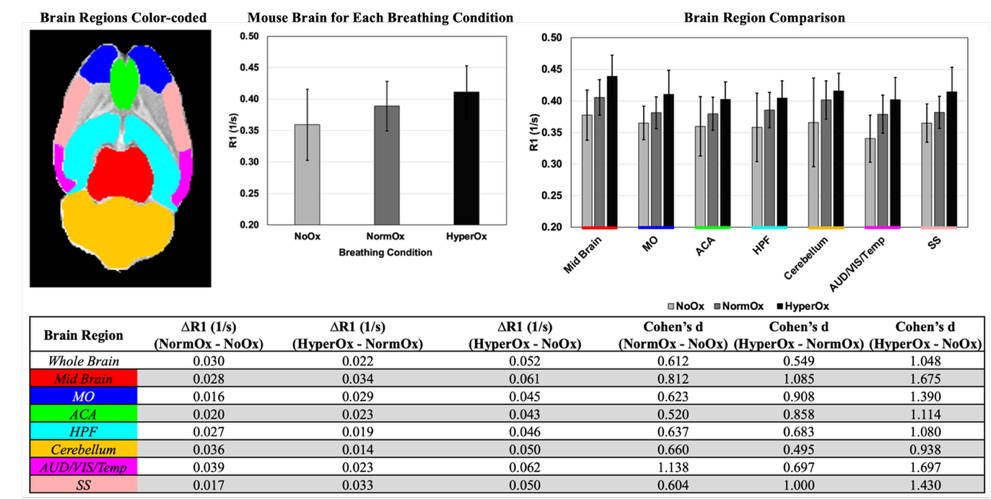


**S.Figure 2:** (A) Upper part of mouse brain with anatomical atlas color-coded. (B) Mean R1 value for the brain for each breathing condition. (C) Mean R1 for each brain region and breathing condition. (D) Table of results from brain regions.

A brain atlas was co-registered to the R_1_ maps to extract mean R_1_ values from seven anatomical regions of interest (ROIs): midbrain, motor cortex (MO), anterior cingulate area (ACA), hippocampal formation (HPF), cerebellum, auditory/visual/temporal cortex (AUD/VIS/Temp), and somatosensory cortex (SS) (**S.Figure 2**). R_1_ increased nearly monotonically across all ROIs from the NoOx to NormOx to HyperOx conditions, consistent with the shortening of T_1_ by paramagnetic molecular oxygen. The whole-brain mean R_1_ increased from 0.359s^-1^ (NoOx) to 0.389s^-1^ (NormOx) to 0.411s^-1^ (HyperOx). Since this experiment was comprised of a
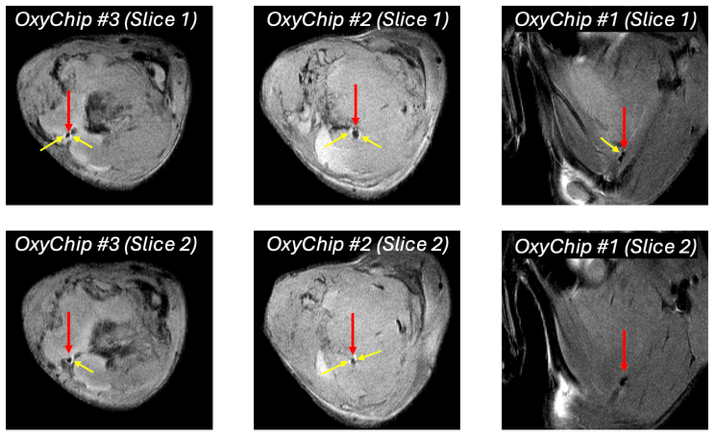
single animal, effect sizes were quantified using Cohen’s d. Cohen’s d was computed from the voxel-wise R_1_ distributions within each ROI using the pooled standard deviation across breathing conditions, to provide a measure of separation between condition-specific R_1_ distributions independent of sample size. The HyperOx-NoOx comparison, representing the largest achievable contrast, showed large effects (d > 0.8) across all ROIs, with the midbrain and auditory/visual/temporal cortexes showing greatest effect (d = 1.675 and 1.697, respectively). The consistency of large effect sizes across anatomically and functionally distinct brain regions suggests that the oxygen sensitivity of the variable-TR RARE sequence is generalizable enough as a contrast mechanism under a hyperoxic challenge. These results provided evidence that the variable-TR RARE relaxometry sequence is sensitive to physiologically relevant changes in tissue oxygenation.

**S.Figure** **3**:T1w images of the OxyChips in tissue. Red arrows identify the OxyChips. Yellow arrows identify the GNP artifacts.

# 3. GNP-induced Susceptibility Artifacts

Gold nanoparticles (GNPs) were incorporated into the OxyChips to facilitate localization within the tissue (**S.Figure 3**). First, the high electron density of gold substantially increases the X-ray attenuation of the chips, rendering them much more visible on a microCT. Second, the magnetic susceptibility mismatch introduced by the GNPs generates localized field inhomogeneities that manifest as susceptibility artifacts primarily along the phase-encoding direction. This produces a partial halo effect in the MR images near the OxyChips which is useful for identifying the chips that would otherwise provide no MR signal. However, this artifact extends into some portions of the immediate surrounding tissue which can elevate apparent R_1_ values. Consequently, R_1_ sampling regions of interest around the OxyChips must be carefully positioned to exclude voxels within the spatial extent of these artifacts to ensure that the measured R_1_ values reflect dissolved oxygen concentration and are not contaminated by the GNP-induced signal enhancement.


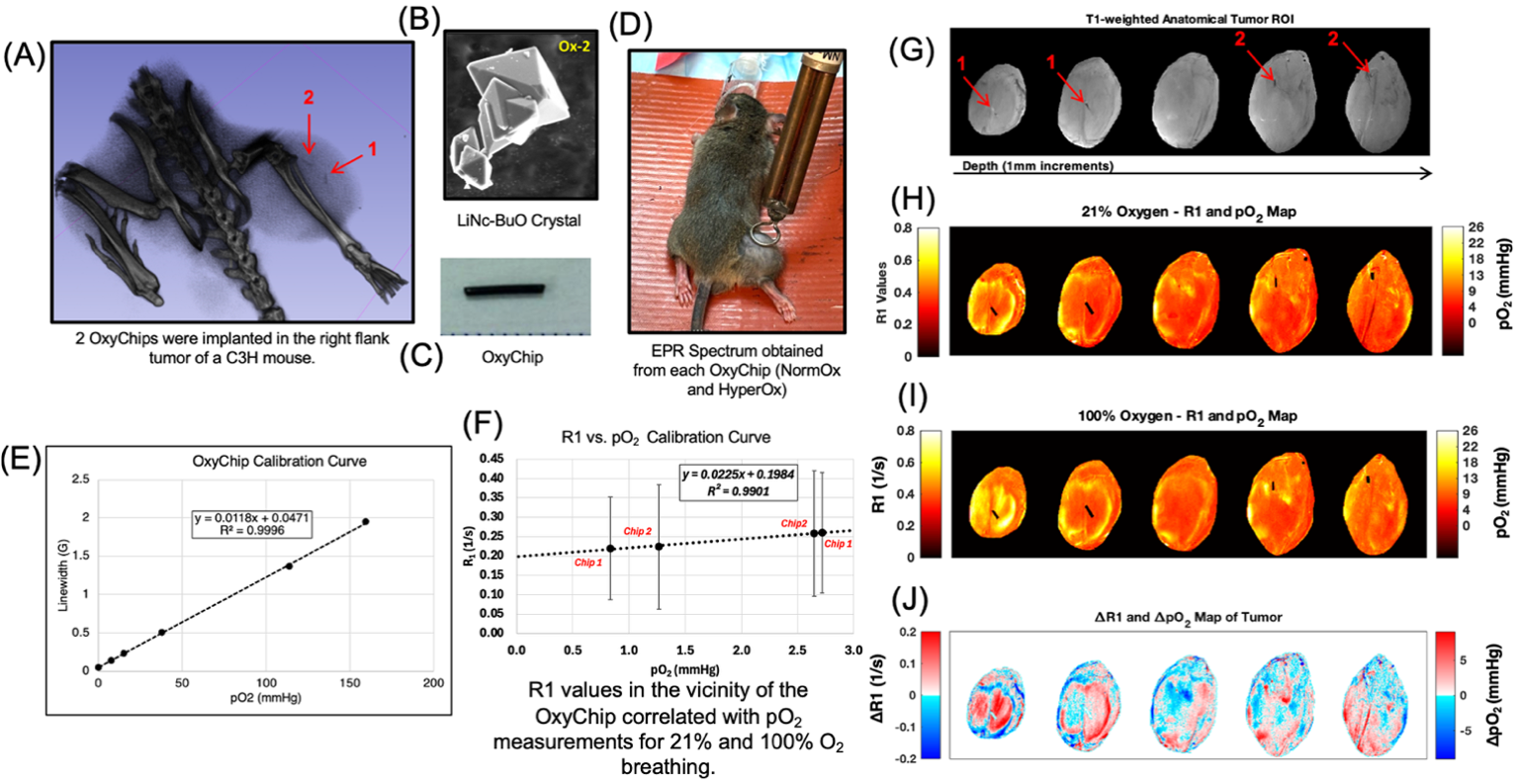


***S.Figure 4****: (A) micro-CT of OxyChip positions. (B) SEM scan of LiNc-BuO crystal. (C) OxyChip. (D) L-band EPR measurement of OxyChips. (E) OxyChip calibration curve. (F) R1 vs pO_2_ calibration curve. (G) T1w image of tumor and OxyChip positions. (H) R1 and pO_2_ maps at 21% O_2_. (I) R1 and pO_2_ maps at 100% O_2_. (J) ∆R1 and ∆pO_2_ maps (100% - 21% O_2_).*

# 4. OxyChip R_1_ Calibration using a Surface Coil, Larger OxyChips, and Reduced Sensor Count

To assess the feasibility of calibrating *in vivo* R_1_ maps using implanted EPR sensors, an experiment was performed in a single male C3H mouse bearing a subcutaneous SCC7 tumor in the right hind flank. Two OxyChips (approximately 2.5 mm x 0.2 mm) were implanted directly into the tumor tissue 48 hours prior to data collection to allow for tissue healing. Three modalities were used: uCT (for OxyChip localization), EPR spectroscopy (pO_2_ measurement), MRI (oxygen-enhanced MRI). MRI data were collected first using a 20-mm Bruker surface coil positioned over the tumor in a 9.4T pre-clinical MRI scanner. An anatomical T_1_-weighted images was first acquired to delineate tumor structure. T_1_ relaxometry mapping was performed under two successive breathing conditions normoxic medical air (NormOx, 21% O_2_) and hyperoxic gas (HyperOx, 100% O_2_) for OE-MRI. A 20-minute equilibration period followed the gas switch before repeating the T_1_ mapping acquisition. T_1_ maps were generated using a variable-TR saturation recovery RARE sequence with the following parameters: TE = 7 ms, TR = [127, 215, 310, 413, 527, 652, 791, 949, 1131, 1346, 1608, 1943, 2409, 3182, and 6000 ms], ETL = 2, AVG = 1, slice thickness = 1 mm, slice gap = 0.3 mm, 6 slices, FOV = 20 × 20 mm², matrix size = 256 × 192, in-plane resolution = 0.078 × 0.104 mm², BW = 318 Hz/px. Each T_1_ mapping acquisition required approximately 35 minutes, yielding a total MRI scan duration of approximately 90 minutes across both conditions inclusive of the gas equilibration period.

This experiment differed from the primary OxyChip calibration approach described in the main manuscript in three respects. First, a surface coil rather than a volume coil was used, introducing B_1_ inhomogeneity across the imaging volume that may contribute to systematic variation in the fitted R_1_ values. Second, the OxyChip sensors employed here were larger than the OxyChips used in the main study, resulting in a larger sensitive volume and correspondingly greater uncertainty in the EPR-derived pO₂ estimates and larger averaging area for the R_1_ map. Third, only two OxyChip sensors were available for calibration rather than three, reducing the degrees of freedom for the R_1_-pO₂ regression and limiting the spatial sampling of the tumor oxygenation landscape. These factors collectively contributed to broader confidence intervals in the resulting calibration relative to the main manuscript protocol, and motivated the methodological refinements described therein.

Within the calibration dataset in the primary manuscript, R_1_ increased linearly with EPR-derived pO_2_ across the measured range (1.0–15.7 mmHg). Ordinary least squares (OLS) regression yielded a slope of 0.0087±0.0016 s^-1^/mmHg, an intercept of 0.301±0.003s^-1^, and an excellent goodness of fit (R^2^ = 0.974, p = 0.0003, RMSE = 0.0075s^-1^), indicating that pO_2_ accounts for approximately 97% of the variance in R_1_ across the calibration range. The supplemental calibration data in **S.Figure 4** produced a steep apparent slope of 0.023 s^-1^/mmHg and a lower intercept of 0.198 s^-1^ (R^2^= 0.990, p = 0.005), both of which differ from the primary calibration. When the two datasets were pooled and fit jointly using OLS, the combined slope was 0.013 s^-1^/mmHg (R^2^ = 0.825, p = 0.0003) (**S.Figure 5**); however, a weighted regression using inverse-variance weights (1/𝜎^2^) to appropriately account for the larger measurement uncertainty in the supplemental data produced a non-significant fit (slope = 0.010 s^-1^/mmHg, p = 0.126), indicating that the two datasets are not consistent with a shared calibration line once measurement uncertainty is accounted for. The slope discrepancy between animals is attributed to two sources: first, B_1_ inhomogeneity inherent to the small surface coil acquisition (20-mm diameter) coupled with the large field strength (9.4T), which introduces spatially varying systematic error in fitted R_1_ values over the imaging volume; and second the larger volume of the OxyChips which increases the spatial averaging as each OxyChip is in contact with more tissue. For these reasons, the protocol in the main manuscript was adapted to use smaller chips (still detectable with L-band *in vivo* EPR), more chips (more calibration points, but still spatially distinguishable to avoid signal overlap), and a custom-built homogeneous volume coil.


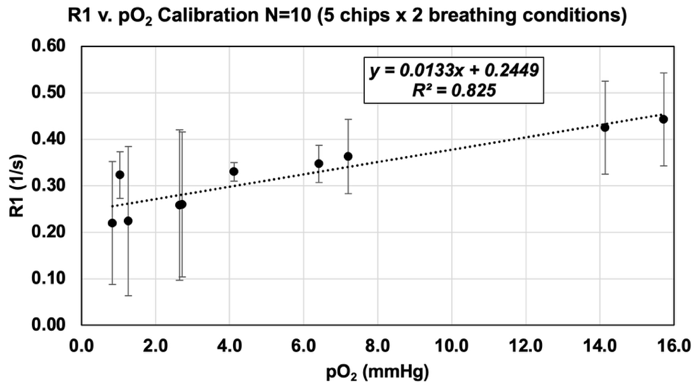


**S.Figure 5**: R_1_-pO_2_ calibration curve from five OxyChips from two mice.
